# Metastasis of Papillary Thyroid Carcinoma to the Maxillary Sinus: Case Report and Review of the Literature

**DOI:** 10.1155/2020/4056901

**Published:** 2020-05-11

**Authors:** Hajime Shimmura, Eri Mori, Rumi Sekine, Masayoshi Tei, Nobuyoshi Otori

**Affiliations:** Department of Otorhinolaryngology, The Jikei University School of Medicine Hospital, 3-19-18 Nishi-shimbashi, Minato-ku, Tokyo, Japan

## Abstract

Metastasis of the thyroid carcinoma to the paranasal sinuses is rarely reported. Among these sinuses, metastasis to the maxillary sinus alone has been reported only in a few cases. This is the first reported case in a 76-year-old woman with papillary thyroid carcinoma metastasizing to the maxillary sinus alone and resected through endoscopic sinonasal surgery. When patients have sinus lesions and a history of malignancy, metastasis should be included in the differential diagnosis. If they have an isolated metastatic lesion to the paranasal sinus, ESS, either palliative or radical, can be a useful treatment option.

## 1. Introduction

Distant metastasis of malignant tumors to the paranasal sinuses accounts for 1.5% of all paranasal sinus carcinomas [1]. The common primary lesions are renal carcinomas [2]. Distant metastasis from the thyroid carcinoma to the paranasal sinuses is rare; only 14 cases have been reported from 1979 to 2018 [3–16]. We report a case of papillary thyroid carcinoma (PTC) (the most frequent type of differentiated carcinoma of the thyroid) metastasizing to the maxillary sinus and present some literature reviews.

## 2. Case Presentation

A 76-year-old woman visited our hospital with a complaint of right intermittent epistaxis that started 2 years ago. The epistaxis gradually worsened several weeks before her visit to our hospital.

She had a medical history of PTC for 8 years and underwent total thyroidectomy, neck dissection twice, stereotactic radiation therapy for metastases to the cervical vertebrae and parapharyngeal space, and radioactive iodine therapy. A year ago, another cervical lymph node metastasis was noted. The surgery had already been scheduled at another hospital when she visited our hospital.

**Figure 1:**
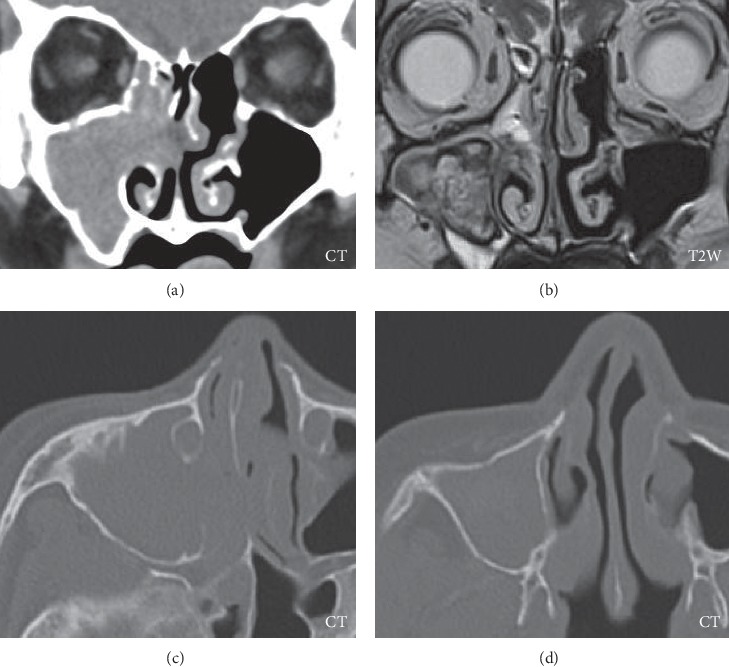
Initial images. CT: shadow of the soft-tissue density of the right maxillary sinus; T2W: well-circumscribed, smoothly marginated tumor with a peripheral low area and a central inhomogeneous area.

Sinonasal endoscopy showed bleeding from the accessory ostium of the right maxillary sinus. Contrast-enhanced computed tomography showed a mass, filling the right maxillary sinus without any angiogenesis, bone destruction, or calcification. Magnetic resonance imaging showed a well-circumscribed and smoothly marginated tumor with a peripheral low area and central inhomogeneous area on T1-and T2-weighted images, primarily suggesting an organized hematoma (Figure 1) [5].

**Figure 2:**
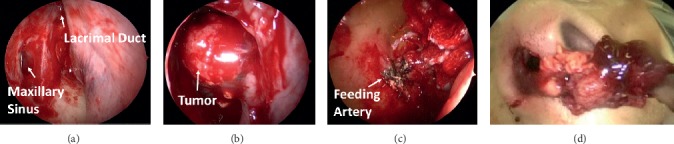
(a) Right maxillary sinus opened with endoscopic modified medial maxillectomy. (b) Tumor (3 × 2 cm) in the right maxillary sinus (with a 70° endoscope). (c) Bleeding from the feeding artery based on the anterior wall of the right maxillary sinus. (d) Feeding artery cauterized and unblocked tumor resected.

**Figure 3:**
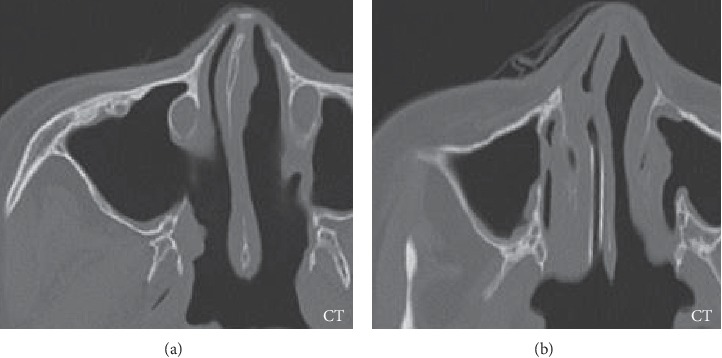
Post-ESS images. CT: Resected tumor in the right maxillary sinus.

**Figure 4:**
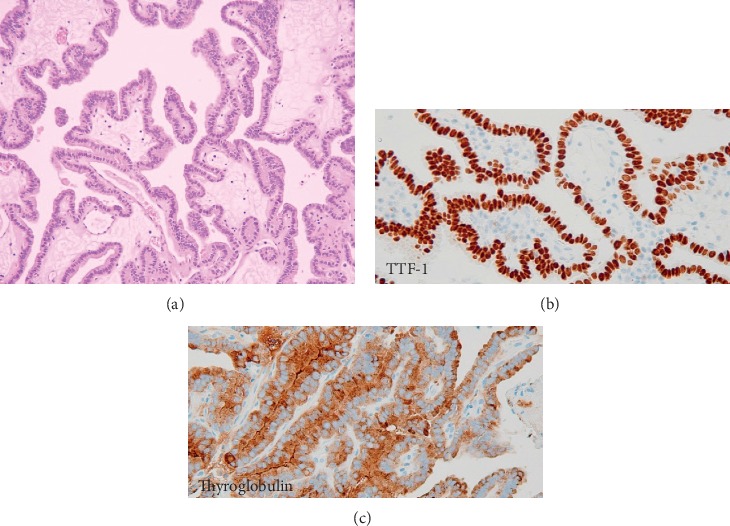
(a) Papillary structure. Cell nuclei are slightly larger with coarsely granular chromatin (hematoxylin and eosin, 100×). (b, c) Immunohistochemical staining revealed tumor cells positive for thyroid transcription factor 1 (TTF-1) (positive for carcinoma arising from follicular epithelial cells) and thyroglobulin.

To stop the bleeding, endoscopic sinonasal surgery (ESS) was performed to examine the tumor and excision. We approached the right maxillary sinus with endoscopic-modified medial maxillectomy. The tumor was approximately 3 × 2 cm with a stem including a feeding artery based on the anterior wall of the right maxillary sinus. We cauterized the feeding artery and resected the tumor en bloc with a mucosa margin of 1 cm around the stem (Figures 2 and 3). Histopathological diagnosis revealed a metastatic PTC tumor to the maxillary sinus (Figure 4). Regarding its subtypes, we did not find any characteristic findings for specific variants type of PTC. We presented the report to her attending oncologist, who discussed with the patient about additional examination and therapy. The decided treatment policy was to undergo planned lymph node resection and no additional examination because of her advanced age and history of total thyroidectomy and radiation therapy. One month after ESS, prescheduled metastatic lymph node resection was performed at another hospital. At 2 years postoperatively, there was no recurrence nor epistaxis.

## 3. Discussion

PTC accounts for 85% of the well-differentiated thyroid carcinomas [17]. It is often slow-growing and localized. The mortality rate of patients with PTC followed up for 16 years was only 6% [18]. Risk factors for recurrence and cancer-related mortality were age at diagnosis, size of the primary tumor, presence of soft-tissue invasion, and distant metastases [18]. The mortality of patients with PTC and its metastases are variable, depending on the site of metastases. The distant metastasis rate is 7–15% in patients with PTC [19, 20]. PTC often shows lymphogenous rather than hematogenous spread [21]. The common sites of distant metastatic lesions of PTC are the lung, bone, and brain in the descending order of frequency [21]. Metastasis of the thyroid carcinoma to the paranasal sinuses is rarely reported.

Immunohistochemical staining of thyroid transcription factor 1 (TTF-1) and thyroglobulin is used in the pathological diagnosis of PTC [21]. It is often used to presume thyroid primary carcinoma at metastatic lesions, as in this case. TTF-1 is a transcription factor involved in the development and differentiation of the thyroid gland and lung. It is expressed in the nucleus of thyroid and lung carcinoma cells [22]. Thyroglobulin originates in the thyroid follicular epithelium. It is utilized as a clinical marker for thyroid follicular epithelial lesions and carcinomas (e.g., papillary carcinoma, follicular carcinoma, and follicular adenoma) [23, 24].

Distant metastasis of malignant tumors to the paranasal sinuses accounts for 1.5% of all paranasal sinus carcinomas [1]. The common primary lesions are renal, lung, breast, thyroid, and prostate carcinomas in the descending order of frequency [2]. The metastatic route depends on the characteristics of the primary tumors. The vertebral plexus is thought to be the main metastatic route, reaching the paranasal sinuses through the intracranial venous plexus [25]. In this case, the presence of tumor metastasis in the cervical spine supports this metastatic pathway. Maxillary sinuses are metastasized the most, followed by the sphenoid, ethmoid, and frontal sinuses [26, 27]. In 77% of cases, metastasis is found in a single paranasal sinus lesion [26]. There are no specific symptoms such as nasal obstruction, epistaxis, proptosis, and diplopia. The treatment may include radioiodine therapy (RI) and radiation therapy [10].

Metastasis of the thyroid carcinomas to the paranasal sinuses is rarely reported worldwide; only 14 cases have been reported from 1979 through 2018 (Table 1). The majority of patients have known thyroid cancer, with a mean age of 56 years. The histologic type of the primary site was mainly follicular thyroid carcinoma (78.6%). Metastases were found mainly in the sphenoid sinus (85.7%) and had mostly spread to multiple sinuses (71.4%). These features are different from those of other metastases of malignant tumors to the paranasal sinuses. The most common symptom is epistaxis, which is attributed to the characteristic vascular metastasizing route of the follicular thyroid carcinoma [28]. This is the first reported case of PTC metastasis to the maxillary sinus alone.

Metastasis of thyroid carcinomas to the maxillary sinus was reported in 6 patients, and the histologic type was follicular carcinoma. Most patients were aged >50 years (66.7%) and predominantly female (83.3%), with a chief complaint of intermittent epistaxis. They were mainly treated with radioisotope therapy, and none of the patients underwent ESS.

The treatment for metastasis of the thyroid carcinomas to the paranasal sinuses is mainly radiation therapy, including RI. In contrast, radical treatment by ESS remains controversial and is mainly used for palliative treatment [29, 30], for example, to relieve pain and control bleeding. An exception is an isolated metastasis case, for which a radical ESS may be a viable option [29]. In this case, metastasis of the thyroid carcinoma was found in the maxillary sinus alone, and with it being an isolated lesion, radical ESS is a useful treatment modality for total resection.

It is an occasionally difficult task for the ESS surgeon to decide the extent of the resection and its surgical margin. Metastatic tumors in the sinuses vary on a case-by-case basis; thus, there are few literature reports on its resection and the relationship between surgical methods and recurrence. In this case, we obtained a surgical margin of 1 cm around the stem of the tumor. We did not suspect the tumor to be malignant preoperatively; hence, we did not perform intraoperative rapid diagnosis. Although it was difficult to diagnose malignancy from preoperative images and metastatic PTCs to nasal sinuses are rare, given the fact that the patient had a medical history of PTC, we should have considered the possibility of malignancy and planned intraoperative rapid diagnosis. A complete curettage of the maxillary mucosa might have been an option, but there is no clear evidence as to whether this option decreases the recurrence rate. Further investigations are required for the surgical method and its margin.

Metastasis of the thyroid carcinoma to the paranasal sinuses is extremely rare, and this is the first reported case of PTC metastasizing to the maxillary sinus alone, which was resected with ESS. ESS can be useful in controlling nasal bleeding due to metastasis of carcinoma to the paranasal sinuses and completely resecting an isolated metastatic lesion. When patients have a sinus lesion and history of malignancy, the differential diagnosis should include metastasis.

## Figures and Tables

**Table 1:** Metastasis of the thyroid carcinomas to the paranasal sinuses.

Author	Year	Age/sex	Pathology type	Metastatic regions	Treatment
Barrs et al. [3]	1979	54 F	Follicular	SS	
Cinberg et al. [4]	1980	80 F	Follicular	MS	
Chang et al. [5]	1983	5 F	Follicular	Papillary, SS, ES, pituitary fossa	
Renner et al. [6]	1984	61 F	Follicular	SS	
Yamasoba et al. [7]	1994	34 F	Follicular	MS, SS, ES, nasal cavity	
Cumberworth et al. [8]	1994	62 F	Follicular	MS, SS, ES	
Freeman et al. [9]	1996	50 M		Papillary, MS, SS, ES, middle cranial fossa	
Altman et al. [10]	1997	81 M	Follicular	SS, ES, clivus	
Argibay [11]	2005	53 F		Papillary, SS, ES	
Nishijima et al. [12]	2010	81 F	Follicular	SS, ES	
Krishnamurthy et al. [13]	2010	31 F	Follicular	MS	RI, rad
Krishnamurthy and Ramshankar [14]	2013	55 F	Follicular	MS, SS, ES	RI, rad
Madan et al. [15]	2013	45 M		Papillary, SS, ES, upper pharynx	RI, rad
Pourseirafi et al. [16]	2014	68 F	Follicular	SS, ES, clivus, orbita	RI
This case	2018	76 F		Papillary, MS	ESS

MS, maxillary sinus; ES, ethmoid sinus; SS, sphenoid sinus; RI, radioiodine therapy; rad, radiation therapy.

## Data Availability

The case data used to support the findings of this study are available from the corresponding author upon request.
